# Curative resection of gallbladder cancer with liver invasion and hepatic metastasis after chemotherapy with gemcitabine plus S-1: report of a case

**DOI:** 10.1186/1477-7819-12-326

**Published:** 2014-11-04

**Authors:** Takashi Okumura, Jun Nakamura, Keita Kai, Yasushi Ide, Hiroaki Nakamura, Hiroki Koga, Takao Ide, Atsushi Miyoshi, Kenji Kitahara, Hirokazu Noshiro

**Affiliations:** Department of Surgery, Saga University Faculty of Medicine, Nabeshima 5-1-1, Saga, 849-8501 Japan; Department of Pathology and Microbiology, Saga University Faculty of Medicine, Nabeshima 5-1-1, Saga, 849-8501 Japan; Oda Hospital, Ohaza Takatsuhara 4360, Kashima City, Saga, 849-1932 Japan

**Keywords:** chemotherapy, gallbladder cancer, gemcitabine, liver metastasis, S-1

## Abstract

A 62-year-old woman diagnosed with gallbladder cancer exhibiting broad liver invasion and metastasis to Couinaud’s hepatic segments 4 and 8 (S4 and S8) consulted her regular doctor. Owing to the presence of liver metastases, she received treatment with gemcitabine plus S-1. After four cycles of chemotherapy, the size of the main lesion dramatically decreased and the two liver metastases disappeared. After six cycles of chemotherapy, the patient was referred to our hospital for surgical treatment. Upon admission, there was no evidence of any distant metastasis, based on a detailed radiological examination. Therefore, we performed cholecystectomy and central bisegmentectomy of the liver after obtaining the patient’s informed consent. Pathological examination demonstrated viable cancer cells with granuloma formation and calcification in the gallbladder, as well as regenerative changes without viable cancer cells in S4 and S8 of the liver. Gemcitabine plus S-1 was again administered as postoperative adjuvant chemotherapy. One and a half years after the surgery, there were no signs of recurrence. In patients selected according to their response to chemotherapy, surgical treatment might therefore be effective against gallbladder cancer with metastasis.

## Background

Although complete surgical resection is the only curative modality for biliary tract cancer, even patients who receive curative resection frequently develop recurrence of the disease [1, 2]. Moreover, no standard chemotherapeutic regimen for advanced biliary tract cancer has been established to date. Recently, combined chemotherapeutic regimens based on gemcitabine, such as gemcitabine plus cisplatin and gemcitabine plus S-1, have become available for patients with unresectable biliary tract cancer [3]. However, the response rates to these regimens range from only 15% to 37%, and the overall survival time is limited to 4.7 to 15.4 months [4]. By contrast, dramatic effects of gemcitabine-based chemotherapy allowing for curative surgery in cases of initially unresectable gallbladder cancer have been reported [5–11]. In this report, we describe our successful treatment of a patient with gallbladder cancer with direct liver infiltration and two hepatic metastases using gemcitabine plus S-1 followed by complete surgical resection.

## Case presentation

**Figure 1:**
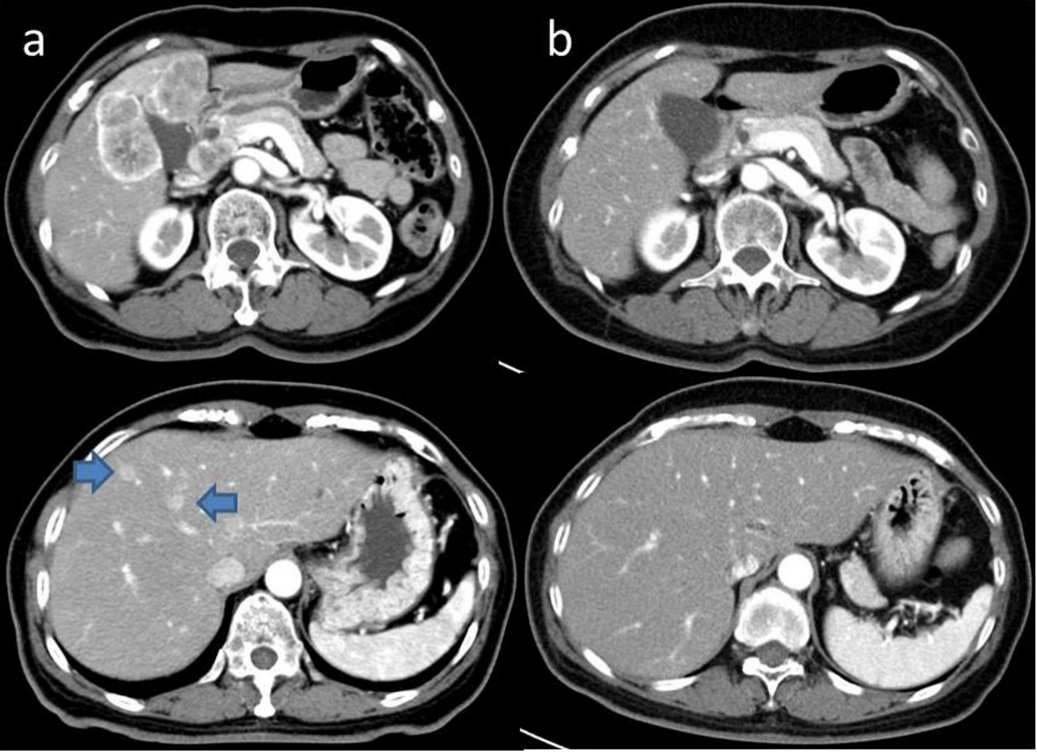
**Changes in CT images of the gallbladder cancer and liver metastases: (a) before chemotherapy; (b) after four cycles of chemotherapy.**

A 62-year-old woman underwent a periodic computed tomography (CT) examination for hepatitis B at another hospital, where she was diagnosed with significantly advanced gallbladder cancer. Her weight was 50.5 kg and her height was 153 cm, and a physical examination revealed no abnormalities. Laboratory tests showed slight elevation of the carbohydrate antigen (CA) 19-9 level at 57 U/ml; however, most of the other results were normal. Enhanced abdominal CT scans obtained on the first admission showed a large mass lesion located in the gallbladder that had invaded the anterior and medial segments of the liver. In addition, two annular enhanced masses in Couinaud’s hepatic segments 4 and 8 (S4 and S8; Figure 1a) were observed on CT examination. The patient was therefore diagnosed as having advanced gallbladder cancer with direct liver invasion and hematogenous metastasis to the liver, T4 N0 M1; stage IVb according to the American Joint Committee on Cancer tumor-node-metastasis (TNM) classification. The disease was judged to be inoperable, owing to the liver metastasis.After obtaining the patient’s informed consent, chemotherapy with gemcitabine plus S-1 was started at a dose of 1,200 mg of gemcitabine once a week for three weeks and 100 mg of S-1 every day for three weeks, followed by one week of rest. Because the patient experienced grade 3 appetite loss, according to the National Cancer Institute common toxicity criteria, the frequency of S-1 administration was reduced to every other day, starting with the second cycle of chemotherapy. This change allowed the patient to continue to receive the treatment as an outpatient. The serum CA19-9 level gradually decreased, and a follow-up CT scan performed after four cycles showed the main lesion to have dramatically reduced in size, while the two liver metastases had completely disappeared (Figure 1b). At that time, we were consulted regarding the feasibility of surgical treatment. We therefore recommended an additional two cycles of chemotherapy, hoping to obtain a further effect and confirm the absence of distant metastasis.

**Figure 2:**
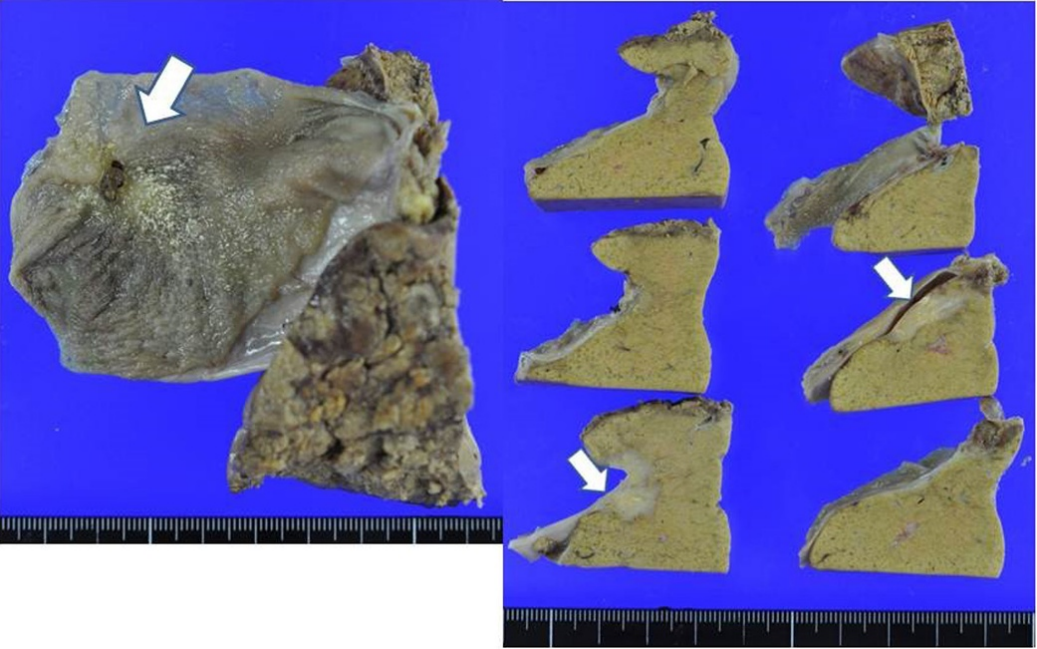
**Macroscopic findings.** Induration of the gallbladder was noted, with solid white lesions in the liver bed.

**Figure 3:**
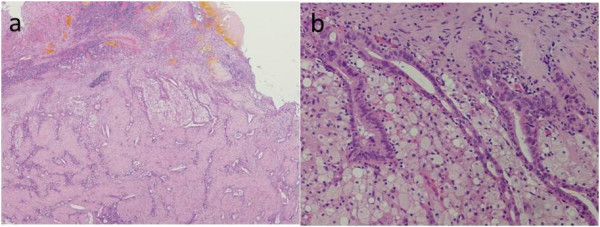
**Microscopic findings.** Viable cancer cells with granuloma formation and calcification were present in the gallbladder, while regenerative changes without viable cancer cells were observed in the liver (S4 and S8). **(a)** H & E staining, ×40; **(b)** H & E staining, ×100.

After the two additional cycles, a CT scan showed that the tumor had further shrunk in size and was detected as only a slightly contrasted lesion in the liver bed. The serum CA19-9 level was 13 U/ml at that time. ^18^ F fluorodeoxyglucose-positron emission tomography (FDG-PET) showed no uptake of FDG in the main lesion or evidence of distant organ metastasis.One month after the final cycle of chemotherapy, we performed cholecystectomy and central bisegmentectomy of the liver with D2 lymph node dissection, which confirmed the absence of both peritoneal dissemination and liver metastasis, based on intraoperative macroscopic and ultrasound findings. Pathological examination demonstrated viable cancer cells (moderately differentiated adenocarcinoma) with granuloma formation and calcification on the free peritoneal side of the gallbladder. The cancer cells were found to have spread widely in the mucosal layer of the gallbladder, although no direct invasion to the liver was observed. Clusters of viable cancer cells were detected in the liver bed apart from the main lesion, diagnosed as liver metastases. In addition, three lymph node metastases were noted on the posterior surface of the pancreatic head. The areas of induration in the S8 segment showed microscopic regenerative changes without viable cancer cells; these findings were histologically compatible with a chemotherapeutic complete response (Figures 2 and 3).

The patient exhibited an uneventful postoperative course. The administration of gemcitabine plus S-1 was restarted as adjuvant chemotherapy and six cycles of the treatment were performed. One and a half years have passed since the operation, with no signs of recurrent disease.

## Discussion

Owing to its anatomical characteristics, gallbladder cancer tends to be diagnosed at an advanced stage, resulting in a poor prognosis. Although only complete surgical resection offers the opportunity for a cure, approximately 70% of patients are ineligible for surgery [2]. Moreover, individuals who do undergo surgical resection often eventually develop recurrence of the disease. Most patients with unresectable or recurrent disease receive palliative chemotherapy, to prolong their survival and relieve symptoms.

In 2009, the ABC-02 trial demonstrated the superiority of the combination of gemcitabine and cisplatin in comparison with gemcitabine alone [12]. In particular, the authors found that the combination regimen significantly prolonged median survival time. In addition, the efficacy of combination chemotherapy using gemcitabine plus S-1 has been evaluated in clinical studies. Three recent phase II studies [4, 13, 14] have established the efficacy of this regimen for unresectable biliary tract cancer, with an overall survival of 11.6 to 12.7 months, a one-year survival rate of 44% to 52%, and a clinical response rate of 32% to 40%. Based on these results, a large randomized phase III study is now planned, to confirm the non-inferiority of gemcitabine plus S-1 combination therapy using a three-week regimen of gemcitabine plus cisplatin combination therapy in Japan [3]. Although the results of this phase III study have not yet been published, we selected gemcitabine plus S-1 combination therapy as first-line chemotherapy in this case, in expectation of its efficacy.

We found that the patient’s two hepatic metastases disappeared after four cycles of chemotherapy after modifying the regimen, owing to grade 3 appetite loss. However, we recommended that the patient receive another two cycles of chemotherapy because we hoped for a further effect and wished to confirm the long-term absence of distant metastasis. To date, there have been no reports regarding the timing of surgery in such cases.

To our knowledge, only eight cases of initially unresectable advanced gallbladder cancer treated with chemotherapy followed by curative surgery have been reported [5–11]. The chemotherapy regimen included gemcitabine alone in two cases, S-1 alone in two cases, gemcitabine plus cisplatin in two cases, and gemcitabine plus S-1 in two cases. The duration of treatment differed in each patient.

With regard to surgical results, one patient died from another disease without progression of gallbladder cancer two months after surgery [5], while the remaining seven patients were each alive without recurrent disease at 10 to 31 months after surgery. The survival data for the patients who underwent resection after chemotherapy appeared to be better than those of the patients with unresectable biliary tract cancer [3]. Moreover, the effects of second-line chemotherapy for biliary tract cancer are unclear [3, 15–24]. The development of effective chemotherapy might allow patients with initially unresectable gallbladder cancer to undergo curative surgery. Surgical treatment should therefore be carefully timed to avoid missing an opportunity for resection in such patients. However, determining the appropriate timing for surgery is difficult and the decision must be made on a case-by-case basis, as surgical methods differ according to the location of the tumor and range of tumor invasion.

In previous reports, the duration of chemotherapy prior to surgery ranged from one to 16 cycles [5–11]. Although surgical strategies (resection of the gallbladder bed, bile duct, and liver, where metastatic disease is originally present before chemotherapy, with or without aggressive lymphadenectomy) for such patients remain controversial, major hepatic resection and reconstruction of the bile duct are often required. Therefore, the indications for and timing of surgery should be carefully determined according to the general condition of the patient. There is no consensus regarding the optimal postoperative adjuvant chemotherapy. We selected gemcitabine plus S-1, since the efficacy of this regimen was confirmed before surgery, performing six cycles of the treatment. However, further research regarding the advantages of postoperative therapy is needed.

## Conclusion

In this case, although the patient was diagnosed with advanced gallbladder cancer with liver metastasis and was not initially considered to be a candidate for surgical resection, gemcitabine plus S-1 chemotherapy was extremely effective and radical surgery was ultimately performed. One and a half years after the operation, there are no signs of recurrence. Although the best strategy for treating advanced gallbladder cancer has not been established, surgical treatment might be effective against gallbladder cancer with metastasis in patients selected according to their response to chemotherapy.

## Consent

Written informed consent was obtained from the patient for the publication of this case report and any accompanying images. A copy of the written consent form is available for review from the editor-in-chief of this journal.

## Abbreviations

CA: carbohydrate antigen
CT: computed tomography
FDG-PET: 18 F fluorodeoxyglucose-positron emission tomography
H & E: hematoxylin and eosin
TNM: tumor-node-metastasis.
